# Localisation inhabituelle de la tuberculose: ostéoarthrite tuberculose du pouce

**DOI:** 10.11604/pamj.2014.19.362.5701

**Published:** 2014-12-09

**Authors:** Aziz Mortaji, Khalid Koulali, Farid Galuia

**Affiliations:** 1 Service d'Orthopédie Traumatologie, Hôpital Militaire Avicenne, Marrakech, Maroc

**Keywords:** Tuberculeuse, ostéoarthrite, pouce, tuberculous, osteoarthritis, thumb

## Abstract

L'ostéoarthrite tuberculose est rare au niveau des doigts. Nous rapportons une observation d'atteinte du pouce chez un patient de 55 ans. Il avait présenté une tuméfaction douloureuse du pouce droit suite à un traumatisme du pouce. L’étude bactériologique et une biopsie avaient permis de confirmer le diagnostic. Un traitement antibacillaire de 12 mois avait donné des résultats satisfaisants. Les particularités de la prise en charge sont discutées par rapport aux données de la littérature.

## Introduction

La tuberculose digitale est rare. Les difficultés du diagnostic sont multiples dues au masque traumatique et la non spécificité des signes cliniques et radiologiques. Nous rapportons un cas d'une ostéoarthrite du pouce découverte suite à un traumatisme.

## Patient et observation

**Figure 1:**
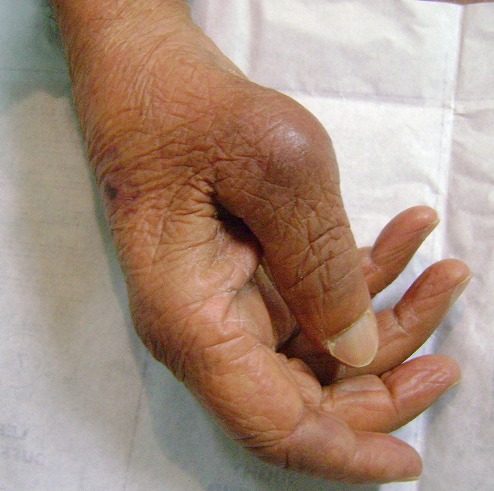
Tuméfaction inflammatoire avec déformation du pouce

Mr M.D, âgé de 55 ans retraité militaire, droitier a consulté pour des douleurs du pouce droit, suite à une chute de sa hauteur sur la main droite. L'examen clinique a objectivé une tuméfaction inflammatoire du pouce droit (Figure 1) avec déformation de la première métacarpophalangienne en flexion et adduction du pouce associé à une limitation douloureuse de sa mobilité. Le reste de l'examen de la main était sans particularité.

**Figure 2:**
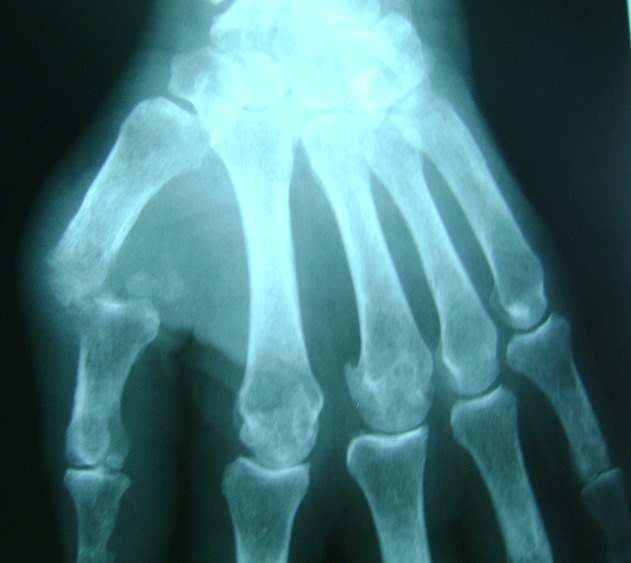
Radiographie de la main montrant une destruction ostéoarticulaire du pouce

Le bilan radiologique standard a révélé une luxation de la première métacarpophalangienne, avec une lyse osseuse de la tête du premier métacarpe (Figure 2). Le bilan biologique a mis en évidence un syndrome inflammatoire avec une VS à 90 mm/heure et une CRP à 75mg/l.

**Figure 3:**
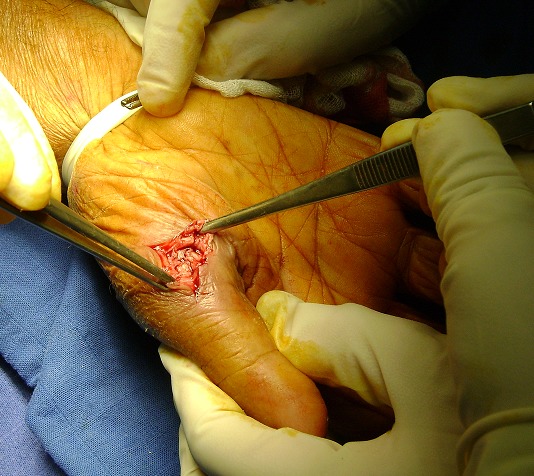
Biopsie chirurgicale directe de la lésion et réduction de la luxation

La biopsie a été réalisée par un mini abord chirurgical direct (Figure 3), suivi d'un curetage du tissu infecté et nécrosé et lavage articulaire. La réduction de la luxation était maintenue à l'aide d'une orthèse du pouce. L'analyse bactériologique était négative notamment la culture sur milieu de Lowenstein. L'examen histologique a objectivé un tissu osseux siège d'un granulome épithélio gigantocellulaire centré par une nécrose caséeuse. Cet aspect caractéristique a permis de poser le diagnostic d'une ostéoarthrite tuberculeuse de la première métacarpophalangienne du pouce.

Le patient a été mis sous polychimiothérapie antibacillaire à base de streptomycine + rifampycine + isoniazide +pyrazinamide pendant 12 mois. Une contention du pouce par une orthèse pendant un mois était associée. L’évolution après un recul de trois ans est satisfaisante avec disparition de la douleur et récupération d'une fonction acceptable du pouce.

## Discussion

La tuberculose ostéoarticulaire sévit encore dans certains pays endémiques, elle vient en 4eme position après la tuberculose pulmonaire, urogénitale et ganglionnaire. La localisation digitale isolée est rare. Elle représente 4 à 8% de l′ensemble des lésions tuberculeuses de tout l′appareil locomoteur. Elle survient préférentiellement chez les enfants entre 1 et 6 ans, ainsi que chez les adultes entre 20 et 50 ans. Il existe une prédominance masculine avec une sex-ratio de 3: 1 [1], notre patient est âgé de 55 ans. La contamination se fait par voie hématogène. La majorité des cas de tuberculose digitale sont primitivement des ostéites longtemps tolérées cliniquement qui s′étendent secondairement à l′articulation [2].

La notion de traumatisme a été rapportée dans certaines séries et serait le mode de révélation d′une lésion préexistante, comme dans notre observation. En effet, cette lésion se caractérise par une discordance radio-clinique du fait de la longue tolérance de la maladie [3]. Elle se manifeste le plus souvent par une tuméfaction longtemps isolée, peu ou pas douloureuse pouvant évoluer vers la fistulisation.

Le bilan inflammatoire et les leucocytes sont souvent normaux. L'intradermoréaction est habituellement positive, mais négative elle n'exclut pas le diagnostic [4]. Radiologiquement, la tuberculose osseuse digitale peut prendre plusieurs aspects, allant d′une simple déminéralisation de la corticale donnant l′aspect d′une ostéoporose,à Une géode,et un pincement articulaire. L′aspect flou, gommé, parfois grignoté de la phalange ainsi que l′interruption de la corticale sont aussi des signes évocateurs. L′absence de réaction périostée caractérise les ostéites tuberculeuses.

La scintigraphie osseuse permet un diagnostic précoce. Elle est pratiquée souvent pour la recherche d'autres localisations de la maladie [5]. Le diagnostic de certitude repose sur l'identification du germe, ce qui est exceptionnel. La biopsie osseuse reste le seul moyen de diagnostic simple et fiable. Qu'elle soit chirurgicale ou percutanée, elle fait le diagnostic dans 9/10 des cas [6]. L'aspect histologique est celui d'un granulome constitué de cellules épithéloïdes et de cellules géantes de Langhans associé à une nécrose caséeuse. Le diagnostic différentiel se fait avec l'ostéomyélite chronique, le chondrome, l'ostéome ostéoïde, la synovite villonodullaire, la sarcoïdose, la maladie de Paget, les hyperparathy- roïdies et la brucellose [7].

Le traitement de la tuberculose osseuse est médical selon le dernier consensus de l'OMS. Il repose sur l'association de plusieurs antituberculeux dont les plus utilisés actuellement sont: rifampicine (R), isoniazide (H), pyrazinamide (Z), éthambutol (E) et streptomycine (S). Le traitement de la tuberculose digitale repose sur les antituberculeux spécifiques pendant une période allant de 6 à 18 mois, avec ou sans immobilisation, seul garant d′une bonne évolution sans séquelles [8].

## Conclusion

La tuberculose ostéoarticulaire du pouce est rare mais doit être évoquée dans les zones d'endémie. Le diagnostic est souvent difficile expliquant la fréquence des formes tardives. Une biopsie chirurgicale permet d'obtenir une confirmation histotologique et aidera à assurer une meilleure efficacité des antituberculeux spécifiques.
